# Linaclotide in Chronic Idiopathic Constipation Patients with Moderate to Severe Abdominal Bloating: A Randomized, Controlled Trial

**DOI:** 10.1371/journal.pone.0134349

**Published:** 2015-07-29

**Authors:** Brian E. Lacy, Ron Schey, Steven J. Shiff, Bernard J. Lavins, Susan M. Fox, Xinwei D. Jia, Rick E. Blakesley, Xinming Hao, Jacquelyn A. Cronin, Mark G. Currie, Caroline B. Kurtz, Jeffrey M. Johnston, Anthony J. Lembo

**Affiliations:** 1 Dartmouth-Hitchcock Medical Center, Lebanon, NH, United States of America; 2 University of Iowa Hospitals and Clinics, Iowa City, IA, United States of America; 3 Forest Laboratories LLC, a subsidiary of Actavis plc, Jersey City, NJ, United States of America; 4 Ironwood Pharmaceuticals, Cambridge, MA, United States of America; 5 Beth Israel Deaconess Medical Center, Boston, MA, United States of America; University Hospital Llandough, UNITED KINGDOM

## Abstract

**Background:**

Abdominal bloating is a common and bothersome symptom of chronic idiopathic constipation. The objective of this trial was to evaluate the efficacy and safety of linaclotide in patients with chronic idiopathic constipation and concomitant moderate-to-severe abdominal bloating.

**Methods:**

This Phase 3b, randomized, double-blind, placebo-controlled clinical trial randomized patients to oral linaclotide (145 or 290 μg) or placebo once daily for 12 weeks. Eligible patients met Rome II criteria for chronic constipation upon entry with an average abdominal bloating score ≥5 (self-assessment: 0 10-point numerical rating scale) during the 14-day baseline period. Patients reported abdominal symptoms (including bloating) and bowel symptoms daily; adverse events were monitored. The primary responder endpoint required patients to have ≥3 complete spontaneous bowel movements/week with an increase of ≥1 from baseline, for ≥9 of 12 weeks. The primary endpoint compared linaclotide 145 μg vs. placebo.

**Results:**

The intent-to-treat population included 483 patients (mean age=47.3 years, female=91.5%, white=67.7%). The primary endpoint was met by 15.7% of linaclotide 145 μg patients vs. 7.6% of placebo patients (*P*<0.05). Both linaclotide doses significantly improved abdominal bloating vs. placebo (*P*<0.05 for all secondary endpoints, controlling for multiplicity). Approximately one-third of linaclotide patients (each group) had ≥50% mean decrease from baseline in abdominal bloating vs. 18% of placebo patients (*P*<0.01). Diarrhea was reported in 6% and 17% of linaclotide 145 and 290 μg patients, respectively, and 2% of placebo patients. AEs resulted in premature discontinuation of 5% and 9% of linaclotide 145 μg and 290 μg patients, respectively, and 6% of placebo patients.

**Conclusions:**

Once-daily linaclotide (145 and 290 μg) significantly improved bowel and abdominal symptoms in chronic idiopathic constipation patients with moderate-to-severe baseline abdominal bloating; in particular, linaclotide significantly improved abdominal bloating compared to placebo, an important finding given the lack of agents available to treat abdominal bloating in chronic idiopathic constipation patients.

**Trial Registration:**

ClinicalTrials.gov NCT01642914

## Introduction

Chronic idiopathic constipation (CIC), estimated to affect between 12% and 19% of North Americans,[1] is characterized by a variety of bowel symptoms including reduced bowel movement (BM) frequency, hard stools, straining during defecation, and a sense of incomplete evacuation after defecation, as well as abdominal symptoms of bloating and discomfort.[2–4] Abdominal bloating refers to subjective sensations of excessive gas, a fullness or tightness in the abdomen, or a feeling of abdominal swelling.[5,6] Subjective sensations of abdominal bloating may or may not be associated with distention, which can be defined as a visible change in abdominal girth.[6] Abdominal bloating is a common and particularly bothersome symptom of CIC.[4,6,7] The etiology of abdominal bloating is incompletely understood, may differ from patient to patient, and is likely multifactorial in nature.[8] Potential causes include visceral hypersensitivity, abnormal intestinal gas transit, impaired evacuation of rectal gas, excess fermentation of bowel contents, an abnormal abdomino-diaphragmatic reflex, and disorders related to the gut microbiota.[6,9] Few treatments have been shown to improve abdominal bloating in patients with CIC.[10,11] In fact, abdominal bloating may be exacerbated by some treatments aimed at improving constipation-related symptoms.[10,11]

Linaclotide is a minimally absorbed 14-amino-acid peptide which binds to and activates guanylate cyclase C (GC-C) on the luminal surface of the intestinal epithelium. Activation of intestinal GC-C receptors results in increased cyclic guanosine monophosphate (cGMP) production, which in turn causes chloride and bicarbonate to be secreted into the gastrointestinal lumen, with consequent increased fluid secretion and accelerated intestinal transit.[12–14] Linaclotide reduces visceral hypersensitivity in animal models, an effect that may be related to cGMP modulation of afferent nerve activity.[15–17] The results of two published Phase 3 trials of linaclotide in CIC patients demonstrated the effectiveness of linaclotide at improving the bowel and abdominal symptoms of CIC in both men and women.[18] Linaclotide is approved in the United States (US), Canada, and Mexico at a dose of 145 μg once daily for the treatment of CIC in adult men and women.

The objective of this Phase 3b clinical trial was to assess the efficacy and safety of linaclotide, at doses of 145 μg and 290 μg administered once daily, in patients with CIC and moderate to severe abdominal bloating at baseline. The hypothesis that linaclotide treatment would lead to an improvement in patient self-assessments of abdominal bloating was of specific interest and will be the key focus of this paper.

## Methods

### Design Overview

This 12-week, multicenter, randomized, double-blind, placebo-controlled, parallel-group, multiple fixed-dose trial was conducted at 141 clinical sites (136 in the United States and 5 in Canada). Recruitment began in August 2012 and ended in February 2013, when the planned sample size was reached. The trial was conducted between 17 August 2012 (first patient enrolled) and 29 May 2013 (last patient completed). The trial was designed, conducted, and reported in compliance with the ethical principles that have their origins in the Declaration of Helsinki and the principles of Good Clinical Practice guidelines. An Institutional Review Board approved the protocol and all trial procedures for all trial centers (Quorum Review IRB, Western Institutional Review Board [WIRB], Mercy Medical Center Institutional Review Board, University of Oklahoma Health Sciences Center Institutional Review Board). All patients gave written informed consent prior to participating in any trial-related procedures. The trial is registered at ClinicalTrials.gov (registration number NCT01642914).

The randomization sequence was generated in SAS by a statistical programmer not otherwise assigned to this trial and the sequence was provided securely to the interactive voice response system (IVRS) vendor. Randomization was blocked by dynamically allocating blocks of 6 to clinical sites as needed; each block contained a random sequence of treatments (145 μg or 290 μg of linaclotide or matching placebo) at a 1:1:1 ratio. Randomization numbers within each block were assigned in ascending order and additional blocks were allocated to clinical sites once complete blocks were used. Trial personnel used the IVRS to randomize patients and to obtain instructions on the study drug to be dispensed.

During an initial screening period of up to 21 days, patients provided blood and urine samples for laboratory testing and were instructed to discontinue any prohibited medications (including laxatives, suppositories, or enemas used to treat CIC). Patients meeting the inclusion and exclusion criteria then entered a 14- to 21-day pretreatment baseline period during which they provided daily and weekly symptom assessments via IVRS. Subsequently, eligible patients were randomized to receive 145 μg or 290 μg of linaclotide or matching placebo (identical appearance and containing the same inactive substances), as an oral capsule administered once daily, for the duration of the 12-week treatment period. Patients were instructed to take the study drug at least 30 minutes before breakfast each day and to make daily IVRS calls to report their symptoms throughout the treatment period.

Trial visits were scheduled at screening, at the start of the baseline period, at randomization (day 1), and throughout the treatment period (weeks 2, 4, 8, and 12 [end-of-trial visit]). All patients and personnel involved in the design and implementation of the trial remained blinded to treatment assignments until the database was locked.

### Trial Patients

Male and female patients were eligible to participate if they were at least 18 years of age and met modified Rome II criteria for chronic constipation.[19] The criteria included < 3 spontaneous bowel movements (SBMs: BMs occurring in the absence of laxative, suppository, or enema use during the preceding 24 hours) per week and at least one of the following symptoms during > 25% of BMs for at least 12 weeks (not necessarily consecutive) within the preceding 12 months: straining, lumpy or hard stools, and a sensation of incomplete evacuation. Additionally, patients needed to report an average of ≤ 6 SBMs and < 3 complete SBMs (CSBMs: SBMs associated with a sensation of complete evacuation) per week, and report an average abdominal bloating score of ≥ 5.0 (self-assessment on a 0–10-point numerical rating scale [NRS], i.e., moderate to severe abdominal bloating) during the 14-day baseline period.

Patients were excluded if they reported loose (mushy) or watery stool (Bristol Stool Form Scale [BSFS] score of 6 or 7) in the absence of laxative, suppository, enema, or other prohibited medication use, for > 25% of BMs during the 12 weeks prior to screening; reported a BSFS score of 6 (loose, mushy stool) for > 1 SBM or 7 (watery stool) for any SBM during the 14-day baseline period; or met the Rome II criteria for irritable bowel syndrome (i.e., abdominal pain or discomfort that is relieved with defecation and/or is associated with a change in stool frequency and/or stool consistency).[20] Other key exclusion criteria included structural GI abnormalities or conditions affecting GI motility; a family history or familial form of colorectal cancer; active peptic ulcer disease; a history of diverticulitis or any chronic condition that could be associated with abdominal pain or discomfort (which could confound the assessments in the trial); a history of fecal impaction requiring hospitalization; or a history of cathartic colon, laxative or enema abuse, ischemic colitis, or pelvic floor dysfunction. Patients were excluded for surgical history reasons including bariatric surgery for treatment of obesity or surgery to remove a segment of the GI tract at any time prior to screening; surgery of the abdomen, pelvis, or retroperitoneal structures during the 6 months prior to screening; appendectomy or cholecystectomy during the 60 days prior to screening; or other major surgery during the 30 days prior to screening.

Colonoscopy requirements were based on the American Gastroenterological Association guidelines current at the time of the trial.[21] Women of childbearing potential were required to use contraceptives and have a negative pregnancy test at the screening and randomization visits. Patients were asked to refrain from making any major lifestyle changes (e.g., starting a new diet or changing their exercise pattern) during the trial. Protocol-defined rescue medication (bisacodyl tablet or suppository) use was not allowed on the day before, day of, or day after the randomization visit. Rescue medications were otherwise allowed during the baseline and treatment periods, when ≥ 72 hours had passed since the patient’s previous BM or when symptoms became intolerable. Patients on a stable dose of fiber, bulk laxatives, or stool softeners during the 30 days prior to screening were allowed to continue, provided they maintained stable dosing throughout the trial.

### Efficacy Assessments and Endpoints

Daily reports by patients via the IVRS included the number of BMs since the previous day’s call and whether rescue medication was used during that time. For each BM, patients reported whether the BM was associated with a sensation of complete emptying (yes/no); stool consistency (7-point BSFS; 1 = “separate hard lumps like nuts [difficult to pass]” to 7 = “watery, no solid pieces [entirely liquid]”); and severity of straining (5-point ordinal scale; 1 = “not at all” to 5 = “an extreme amount”). Daily patient reports also included severity of abdominal bloating, abdominal discomfort, abdominal pain at its worst, abdominal cramping, and abdominal fullness (all rated by the patient on a 0–10-point NRS where a higher score indicated greater severity) during the previous 24 hours.

Weekly IVRS assessments included constipation severity (5-point ordinal scale; 1 = “none” to 5 = “very severe”), adequate relief of constipation symptoms (yes/no), and degree of relief of constipation symptoms (7-point balanced scale; 1 = “completely relieved” to 7 = “as bad as I can imagine”).

#### Primary endpoint

The primary endpoint of the trial defined a responder as a patient who had ≥ 3 CSBMs per week and an increase of ≥ 1 CSBM per week from baseline, in the same week, for at least 9 of the 12 weeks of the treatment period (9/12 week CSBM 3+1 responder), for the linaclotide 145 μg group (the approved dose for the treatment of CIC) compared to the placebo group.

#### Secondary and additional endpoints

Secondary endpoints included an analysis of the primary endpoint parameter for the linaclotide 290 μg group versus placebo, and abdominal bloating change-from-baseline and responder endpoints for both linaclotide dose groups versus placebo. Other secondary endpoints compared the linaclotide 145 μg group to the placebo group; these parameters were assessed as additional endpoints comparing the linaclotide 290 μg group to the placebo group. All endpoints presented were pre-specified. A number of endpoints were updated while the trial was ongoing (and data were still blinded) to ensure consistency with the approved Linzess label and subsequent feedback from the Food and Drug Administration.

Secondary endpoints included 12-week average change from baseline in stool frequency (SBM and CSBM frequency rate), stool consistency, straining, abdominal bloating, and days per week with an SBM; and 12-week percent change from baseline in abdominal bloating; as well as weekly change from baseline in SBM and CSBM frequency rate (at weeks 1, 4, 8, and 12), stool consistency and straining (at week 12), and distribution of percent change from baseline in abdominal bloating (at week 12). Secondary responder endpoints included an analysis of the primary endpoint parameter (9/12 week CSBM 3+1 responder) for the linaclotide 290 μg group versus placebo, 9/12 week mild straining and diarrhea-free responder (non-missing weekly average straining score ≤ 2 and no diarrhea AEs, in the same week, for at least 9 of the 12 weeks of the treatment period), 6/12 week abdominal bloating 30% responder (improvement in weekly abdominal bloating score of ≥ 30% from baseline for at least 6 of the 12 weeks of the treatment period), and SBM within 24 hours responder (≥ 1 SBM within 24 hours of the first dose of investigational product). The time to first SBM (number of hours from the time of first dose of investigational product to the time of the first SBM) was also assessed as a secondary endpoint.

A number of additional endpoints were also assessed in both linaclotide dose groups versus placebo, including CSBM responder parameters; other abdominal bloating responder parameters; weekly percent change from baseline in abdominal bloating; 12-week change-from-baseline parameters for abdominal symptoms (discomfort, pain, cramping, and fullness) and for constipation severity; 12-week percent change from baseline in abdominal discomfort and pain; incremental improvement in abdominal bloating; adequate relief responders, and percent of patients who had an increase from baseline in the percentage of days they used of rescue medication.

### Safety Assessments

The clinical site investigator evaluated all patient-reported treatment-emergent adverse events (AEs) and serious adverse events (SAEs) at each scheduled trial visit, and assessed each event for severity and relationship to the blinded trial treatment. Other safety evaluations included physical examinations, vital sign measurements, and blood and urine sample collection for analysis (at screening and weeks 1, 4, and 12).

### Statistical Analysis

This trial was powered based on the primary endpoint (i.e., 9/12 week CSBM 3+1 responder). On the basis of results from two previous Phase 3 CIC trials,[18] a sample size of 450 patients (150 patients per treatment group) was determined to provide 92% power to detect a difference in the primary endpoint between placebo and the linaclotide 145 μg group. The overall family-wise type I error rate for testing the primary and secondary endpoints was controlled at the 0.05 significance level using a 4-step serial gatekeeping multiple comparisons procedure. Statistical analyses were performed using version 9.3 of SAS on the Linux operating system.

Responder endpoints were analyzed using a Cochran-Mantel-Haenszel (CMH) test controlling for geographic region; patients who were not assessable for responder endpoints due to missing information were considered non-responders. Continuous change-from-baseline and percent-change-from-baseline endpoints were analyzed using an analysis of covariance (ANCOVA) model with fixed-effect terms for treatment group and geographic region and the corresponding baseline value as a covariate; the distribution of the percent change from baseline in abdominal bloating at week 12 for each of the two linaclotide dose groups was compared to that of the placebo group using a Kolmogorov-Smirnov test. Means presented are the least-squares means (i.e., means adjusted for the other effects) from the ANCOVA model. Due to the potential for trial centers to have a small number of patients, geographic region was used as a factor in the analyses rather than trial center. For the time-to-event parameter, the time-to-event distribution for each of the two linaclotide dose groups was compared with that of the placebo group using a log-rank test stratified by geographic region.

All randomized patients who received at least one dose of study drug were included in the safety analyses (safety population). Efficacy analyses were based on the intent-to-treat (ITT) population, which included all patients in the safety population who had at least one post-randomization entry of the primary efficacy assessment (i.e., the daily IVRS assessment of CSBMs).

## Results

### Patient Disposition, Demographics, and Baseline Characteristics

**Fig 1:**
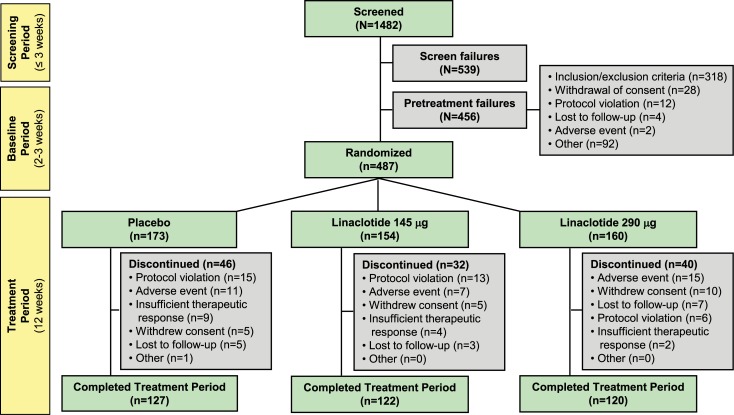
Patient Flow Through the Trial. Screen failures who were rescreened and failed a second time during the screening period were counted once as screen failures. Screen failures who were rescreened, entered the baseline period, and then failed during the baseline period were counted once as pretreatment failures.

Of the 1482 patients who were screened for participation in this trial, 487 patients were randomized to treatment (Fig 1). A total of 486 patients received at least one dose of study drug and were included in the safety population (153 linaclotide 145 μg, 160 linaclotide 290 μg, and 173 placebo patients); of those patients, 483 patients had at least one post-randomization entry of the primary efficacy assessment and were included in the ITT population (153 linaclotide 145 μg, 159 linaclotide 290 μg, and 171 placebo patients). A total of 369 patients (76% of patients randomized) completed the 12-week treatment period. The treatment groups were well-balanced with regard to demographics and baseline constipation symptoms (Table 1). During the pretreatment baseline period, approximately 79% of patients had no CSBMs and the mean abdominal bloating score was 7.1 on a 0–10-point NRS. Mean compliance with study-drug dosing (assessed by counting pills returned at study visits) up to the time of discontinuation or completion of the 12-week treatment period was 98% and 97% in the linaclotide 145 μg and 290 μg groups, respectively, and 98% in the placebo group. Use of fiber, bulk laxatives, or stool softeners was low during the trial (<5% of patients) and was similar across treatment groups.

**Table 1:** Summary of Patient Demographic and Baseline Characteristics (ITT Population).

	Placebo	Linaclotide	
		145 μg	290 μg
	(N = 171)	(N = 153)	(N = 159)
**Demographic data**			
Age (years), mean (range)	46.4 (18–78)	48.3 (20–77)	47.4 (19–80)
≥ 65 years, n (%)	16 (9.4)	12 (7.8)	18 (11.3)
Sex, n (%)			
Female	157 (91.8)	138 (90.2)	147 (92.5)
Male	14 (8.2)	15 (9.8)	12 (7.5)
Race, n (%)			
White	119 (69.6)	97 (63.4)	111 (69.8)
Black	46 (26.9)	54 (35.3)	47 (29.6)
Other	6 (3.5)	2 (1.3)	1 (0.6)
BMI (kg/m^2^), mean (SD)	28.4 (6.1)	29.2 (6.0)	29.8 (6.6)
**Baseline data, mean (SD)**			
Abdominal bloating ^a^	7.1 (1.2)	7.1 (1.3)	7.1 (1.3)
CSBMs/week	0.2 (0.4)	0.2 (0.4)	0.2 (0.4)
SBMs/week	1.8 (1.2)	1.7 (1.2)	1.6 (1.4)
Days with an SBM (per week)	1.7 (1.1)	1.6 (1.1)	1.5 (1.2)
Stool consistency score ^b^	2.3 (1.1)	2.4 (1.0)	2.3 (1.0)
Straining score ^c^	3.6 (0.8)	3.7 (0.8)	3.6 (0.9)
Constipation severity ^d^	3.9 (0.6)	3.9 (0.6)	3.9 (0.7)

BMI = body mass index; CSBM = complete spontaneous bowel movement; ITT = intent-to-treat; SBM = spontaneous bowel movement; SD = standard deviation.

^a^ Assessed using an 11-point numerical rating scale (0 to 10; higher score indicates greater severity).

^b^ Assessed using the 7-point BSFS (1 = separate hard lumps like nuts to 7 = watery, no solid pieces).

^c^ Assessed using a 5-point ordinal scale (1 = not at all to 5 = an extreme amount).

^d^ Assessed using a 5-point ordinal scale (1 = none to 5 = very severe).

### Efficacy

For all primary and secondary efficacy endpoints, the linaclotide groups demonstrated statistically significant improvement compared with the placebo group (controlling for multiplicity).

**Fig 2:**
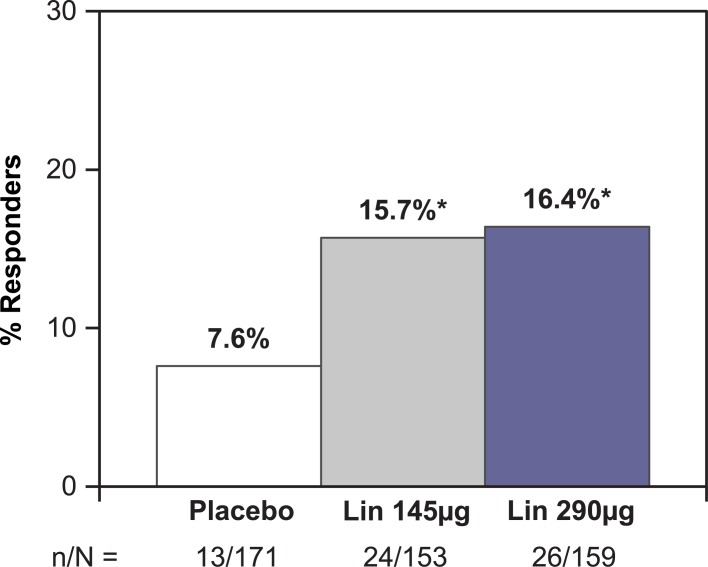
Primary Endpoint. Intent-to-treat Population; Responder = patient who had ≥ 3 CSBMs and an increase of ≥ 1 CSBM from baseline, in the same week, for at least 9 of the 12 treatment-period weeks. Note: Primary endpoint for linaclotide 145 μg vs. placebo; secondary endpoint for linaclotide 290 μg vs. placebo. CSBM = complete spontaneous bowel movement; ITT = intent to treat; Lin = linaclotide; n = number of patients meeting the responder endpoint; N = number of patients in the ITT population. * P < 0.05; P values were obtained from a Cochran-Mantel-Haenszel test controlling for geographic region, comparing each linaclotide dose vs. placebo in a pairwise manner.

A total of 15.7% (24 of 153 patients) in the linaclotide 145 μg group met the responder requirements of the primary endpoint, compared with 7.6% (13 of 171 patients) in the placebo group (*P* = 0.0264; odds ratio 2.23; 95% CI [1.09, 4.56]; Fig 2 and Table 2). For the corresponding analysis in the linaclotide 290 μg group, which was a secondary endpoint, 16.4% (26 of 159 patients) met the responder requirements (*P* = 0.0109 versus placebo; odds ratio 2.45; 95% CI [1.21, 4.96]). The number needed to treat (NNT) for the primary endpoint (in the linaclotide 145 μg group) was 12.4, and for the corresponding secondary endpoint (in the linaclotide 290 μg group) was 11.4. When the components of the primary endpoint were considered for the linaclotide 145 μg group, 15.7% of patients had ≥ 3 CSBMs per week for at least 9 of 12 weeks, compared with 8.2% in the placebo group (*P* = 0.0413); and 26.8% of patients in the linaclotide 145 μg group had an increase from baseline of ≥ 1 CSBM per week for at least 9 of 12 weeks, compared with 16.4% in the placebo group (*P* = 0.0243).

**Table 2:** Efficacy Results During the 12-week Treatment Period (ITT Population).

	Placebo	Linaclotide			
		145 μg		290 μg	
	(N = 171)	(N = 153)	*P* value	(N = 159)	*P* value
**Primary Endpoint**					
% of patients with ≥ 3 CSBMs/week and increase of ≥ 1 CSBM/week for ≥ 9/12 weeks ^c^	7.6	15.7	0.0264***	16.4	0.0109**
**Abdominal Bloating**					
Mean abdominal bloating score (11-point NRS) ^a^	5.5	4.7		4.6	
Change from baseline, mean ^a^ ^,^ ^b^	-1.6	-2.5	0.0002**	-2.5	0.0002**
% change from baseline, mean ^a^ ^,^ ^b^	-22.7	-34.9	0.0004**	-34.3	0.0006**
% change from baseline at week 12, mean ^b^	-30.9	-44.9	0.0030*	-46.5	0.0011*
% of patients with ≥ 30% weekly mean decrease in abdominal bloating for ≥ 6/12 weeks ^c^	29.2	40.5	0.0324**	43.4	0.0083**
% of patients with ≥ 50% weekly mean decrease in abdominal bloating for ≥ 6/12 weeks ^c^	18.1	28.8	0.0215*	29.6	0.0160*
% of patients with ≥ 30% mean decrease in abdominal bloating ^a^ ^,^ ^c^	39.2	51.0	0.0313*	53.5	0.0104*
% of patients with ≥ 50% mean decrease in abdominal bloating ^a^ ^,^ ^c^	17.5	31.4	0.0035*	33.3	0.0011*
% of patients with ≥ 1-point mean decrease in abdominal bloating ^a^ ^,^ ^c^	49.7	69.9	0.0002*	67.3	0.0012*
Change from baseline in % of days with abdominal bloating < 5, mean ^a^ ^,^ ^d^	26.7	37.7	0.0062*	39.7	0.0006*
**CSBMs**					
Mean CSBMs/week ^a^	1.2	2.5		2.5	
Change from baseline in CSBMs/week, mean ^a^ ^,^ ^b^	1.0	2.3	<0.0001**	2.3	<0.0001*
% of patients with ≥ 3 CSBMs/week for ≥ 9/12 weeks ^c^	8.2	15.7	0.0413*	16.4	0.0178*
% of patients with increase of ≥ 1 CSBM/week for ≥ 9/12 weeks ^c^	16.4	26.8	0.0243*	29.6	0.0045*
**SBMs**					
Mean SBMs/week ^a^	3.3	5.2		5.2	
Change from baseline in SBMs/week, mean ^a^ ^,^ ^b^	1.6	3.6	<0.0001**	3.6	<0.0001*
Change from baseline in days/week with an SBM, mean ^a^ ^,^ ^b^	1.2	2.3	<0.0001**	2.1	<0.0001*
SBM ≤ 24 hours after first dose, % ^c^	42.1	61.4	0.0006**	59.1	0.0022*
Time to first SBM after first dose, median number of hours ^e^	28.1	12.5	0.0054**	19.4	0.0470*
**Stool Consistency**					
Mean BSFS score (1–7) ^a^	3.1	4.3		4.6	
Change from baseline, mean ^a^ ^,^ ^b^	0.7	1.9	<0.0001**	2.3	<0.0001*
**Straining**					
Mean straining score (1–5) ^a^	2.8	2.2		2.1	
Change from baseline, mean ^a^ ^,^ ^b^	-0.8	-1.5	<0.0001**	-1.5	<0.0001*
% of patients with mean weekly straining score ≤ 2 *and* no diarrhea AEs for ≥ 9/12 weeks ^c^	8.8	24.8	0.0001**	17.0	0.0299*
**Constipation Severity**					
Mean constipation severity score (1–5) ^a^	3.1	2.7		2.6	
Change from baseline, mean ^a^ ^,^ ^b^	-0.8	-1.3	<0.0001*	-1.4	<0.0001*
**Adequate Relief of CIC Symptoms**					
% of patients reporting adequate relief for ≥ 6/12 weeks ^c^	27.5	49.0	<0.0001*	50.9	<0.0001*
**Rescue Medication Use**					
% of patients with an increase in the percentage of days using rescue medication^c^	32.7	17.6	0.0018*	13.8	<0.0001*

ANCOVA = analysis of covariance; BSFS = Bristol Stool Form Scale; CIC = chronic idiopathic constipation; CSBM = complete SBM; ITT = intent to treat; NRS = numerical rating scale; SBM = spontaneous bowel movement.

*** Primary endpoint (nominal *P* value)

** Secondary endpoint (nominal *P* value)

* Additional endpoint

Note: For all primary and secondary efficacy endpoints, the linaclotide groups demonstrated statistically significant improvement compared with the placebo group, controlling for multiplicity.

^a^ Means are over the 12-week treatment period.

^b^ Changes from baseline are the least-squares means from an ANCOVA model; *P* values were based on a comparison of linaclotide vs. placebo using an ANCOVA model with treatment group and geographic region as factors and corresponding baseline value as a covariate.

^c^ *P* values were based on a comparison of linaclotide vs. placebo using a Cochran-Mantel-Haenszel test controlling for geographic region.

^d^ Changes from baseline are the arithmetic means; *P* values were based on a comparison of linaclotide vs. placebo using rank-transformed normal scores in an ANCOVA model with treatment group and geographic region as factors and corresponding baseline value as a covariate.

^e^ *P* values were based on a comparison of linaclotide vs. placebo time-to-event distributions using log-rank test stratified by geographic region.

**Fig 3:**
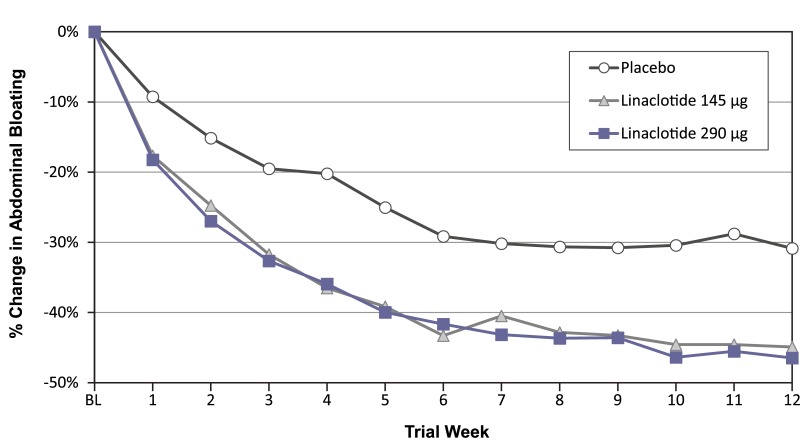
Percent Change from Baseline in Abdominal Bloating by Week. Intent-to-treat Population; % change from baseline in abdominal bloating during each week of the treatment period. % changes from baseline are the least-squares means from an analysis of covariance (ANCOVA) model. Note: % change from baseline in abdominal bloating at each treatment period week was an additional endpoint for both linaclotide dose groups vs. placebo. Both linaclotide doses are associated with greater improvement than placebo at all individual treatment weeks (all reported individual *P* values < 0.05 for both linaclotide groups vs. placebo; *P* values were obtained from an ANCOVA model with treatment group and geographic region as factors and baseline abdominal bloating score as a covariate).

**Fig 4:**
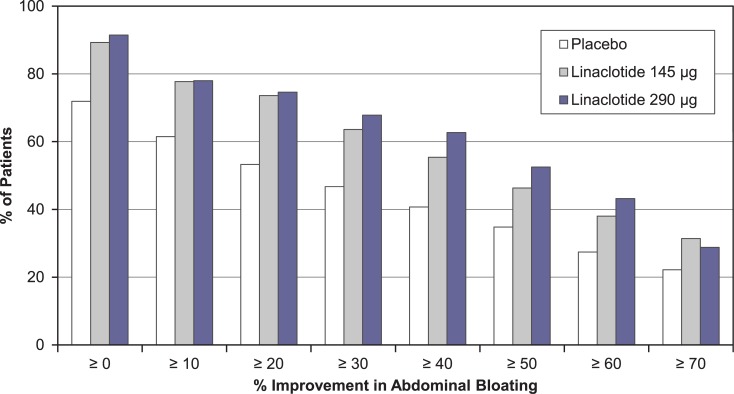
Incremental Percent Improvement in Abdominal Bloating at Week 12. Intent-to-treat Population; % improvement in abdominal bloating at week 12 (end of the treatment period). Note: The distribution of % improvement in abdominal bloating at week 12 was a secondary endpoint for both linaclotide dose groups. (*P* < 0.01 for both linaclotide groups vs. placebo; *P* values were obtained from a Kolmogorov-Smirnov test for equality of distribution.)

Both the 145 μg and 290 μg linaclotide dose groups had significantly greater mean changes from baseline and responder rates for secondary abdominal bloating endpoints compared to placebo (Table 2). Mean percent improvement from baseline in abdominal bloating over the 12-week treatment period was 34.9% and 34.3% in the linaclotide 145 μg and 290 μg groups, respectively, compared with 22.7% in the placebo group (*P* < 0.001; Table 2). A separation between the linaclotide groups and the placebo group in percent change from baseline in abdominal bloating was seen beginning at week 1 and continued throughout the duration of the treatment period (Fig 3). Likewise, both the 145 μg and 290 μg linaclotide doses were superior to placebo when considering the distribution of the percent change from baseline in abdominal bloating at week 12, as illustrated by the incremental mean percent improvements from ≥ 0% to ≥ 70% (*P* < 0.01; Fig 4). Approximately one-third of linaclotide-treated patients (31.4% and 33.3% in the 145 μg and 290 μg groups, respectively) had a mean decrease from baseline in abdominal bloating of ≥ 50% over the 12-week treatment period (additional efficacy endpoint), compared with 17.5% of patients in the placebo group (*P* < 0.01; Table 2).

For all secondary endpoints related to bowel symptoms (SBM and CSBM frequency, stool consistency, and straining), linaclotide-treated patients experienced significantly greater change-from-baseline improvements and responder rates compared with patients in the placebo group (*P* < 0.05; Table 2 and S1 Table). Linaclotide-treated patients at both dose levels experienced shorter times from first dose of study drug to first SBM compared with placebo-treated patients, with median times of 12.5 hours and 19.4 hours in the 145 μg and 290 μg dose groups, respectively, versus 28.1 hours in the placebo group (*P* < 0.05 for comparison of time-to-event distributions; Table 2). Additional global measures (including constipation severity and adequate relief of CIC symptoms) were also improved in linaclotide-treated patients compared with placebo-treated patients (*P* < 0.0001; Table 2). Other efficacy results are summarized in S1 Table.

### Safety

A total of 75 patients (49.0%) in the linaclotide 145 μg group, 76 patients (47.5%) in the linaclotide 290 μg group, and 65 patients (37.6%) in the placebo group reported at least one AE during the 12-week treatment period (Table 3). Most AEs were mild or moderate in severity (139 of 152 AEs [91.4%] linaclotide 145 μg; 142 of 148 AEs [95.9%] linaclotide 290 μg; 126 of 126 AEs [100%] placebo). AEs resulted in the premature discontinuation of 7 patients (4.6%) in the linaclotide 145 μg group, 15 patients (9.4%) in the linaclotide 290 μg group, and 11 patients (6.4%) in the placebo group.

**Table 3:** Adverse Events During the Treatment Period (Safety Population).

Adverse event (preferred term)	Placebo	Linaclotide	
		145 μg	290 μg
	(N = 173)	(N = 153)	(N = 160)
	n (%)	n (%)	n (%)
Patients with at least one AE	65 (37.6)	75 (49.0)	76 (47.5)
Diarrhea	4 (2.3)	9 (5.9)	27 (16.9)
Upper respiratory tract infection	5 (2.9)	9 (5.9)	5 (3.1)
Nausea	3 (1.7)	6 (3.9)	7 (4.4)
Nasopharyngitis	5 (2.9)	5 (3.3)	6 (3.8)
Sinusitis	3 (1.7)	9 (5.9)	2 (1.3)
Influenza	1 (0.6)	3 (2.0)	7 (4.4)
Abdominal pain	2 (1.2)	6 (3.9)	3 (1.9)
Urinary tract infection	3 (1.7)	6 (3.9)	3 (1.9)
Vomiting	1 (0.6)	6 (3.9)	2 (1.3)
Bronchitis	4 (2.3)	3 (2.0)	4 (2.5)
Back pain	1 (0.6)	2 (1.3)	4 (2.5)

AE = adverse event; n = number of patients with AE (patients were counted only once within each preferred term) AEs reported in ≥ 2% of patients in either linaclotide group and at an incidence greater (in either linaclotide group) than reported in the placebo group.

The most common AE was diarrhea, which was reported by 9 patients (5.9%) and 27 patients (16.9%) in the linaclotide 145 μg and 290 μg groups, respectively, compared with 4 patients (2.3%) in the placebo group (Table 3). The occurrences of diarrhea were reported to be mild or moderate in 7 of 9 patients (77.8%) in the linaclotide 145 μg group, 25 of 27 patients (92.6%) in the linaclotide 290 μg group, and all (4 of 4) of the patients in the placebo group. Of the patients who developed diarrhea, 4 of 9 patients (44.4%) in the linaclotide 145 μg group and 12 of 27 patients (44.4%) in the linaclotide 290 μg group had onset within the first week of treatment, compared with 0 of 4 patients in the placebo group; the median time to first onset of diarrhea was 11 days and 8 days post-first-dose in the linaclotide 145 μg and 290 μg groups, respectively, compared with 25 days in the placebo group. No SAEs of diarrhea were reported during the trial. No clinically significant sequelae (e.g., orthostatic hypotension or dehydration) or occurrences of potentially clinically significant vital signs or laboratory values for sodium, potassium, blood urea nitrogen, or creatinine were reported in patients with diarrhea. Diarrhea was the most common AE leading to treatment discontinuation in linaclotide-treated patients (2 patients [1.3%] and 8 patients [5.0%] in the 145 μg and 290 μg groups, respectively, vs. 1 patient [0.6%] in the placebo group).

Serious adverse events (SAEs) were reported by 4 patients (2.6%) in the linaclotide 145 μg group, 2 patients (1.3%) in the linaclotide 290 μg group, and 2 patients (1.2%) in the placebo group. SAEs reported in patients treated with linaclotide 145 μg were abdominal pain, *Clostridium difficile* colitis, non-cardiac chest pain, and uterine leiomyoma; and SAEs reported in patients treated with linaclotide 290 μg were anemia, viral gastroenteritis, pneumonia, and upper respiratory tract infection. SAEs reported in placebo patients were abdominal pain and brain stem infarction. None of the SAEs were considered by the investigators to be treatment-related. There were no deaths reported in this trial.

There were no clinically significant differences between the linaclotide groups and the placebo group in the incidence of abnormal laboratory parameters or vital signs.

## Discussion

This 12-week, randomized, double—blind, placebo-controlled clinical trial in CIC patients with moderate to severe abdominal bloating confirms the results observed in the two earlier phase 3 CIC trials of linaclotide, including the significant effect of linaclotide on improving the bowel symptoms of CIC.[18] In all 3 trials, a significantly greater proportion of linaclotide-treated patients met the requirements of the primary endpoint (responder defined as 3 or more CSBMs per week and an increase of at least 1 CSBM per week for at least 9 of 12 weeks) compared with placebo-treated patients (*P* < 0.05 in all 3 trials).[18] Furthermore, this trial provides new and important data on the effect of linaclotide on abdominal bloating in CIC.

Abdominal bloating is present in up to 90% of patients with CIC and is often reported as the most bothersome symptom of constipation.[6,7] This is the first published trial to evaluate the effects of a treatment on CIC patients with moderate to severe baseline abdominal bloating. The abdominal bloating data were evaluated in several ways, including both change-from-baseline assessments and responder analyses. In this trial of CIC patients with moderate to severe baseline abdominal bloating (i.e., mean of ≥ 5 on a 0–10 scale, as assessed by the patient), linaclotide improved abdominal bloating with a ≥45% reduction in bloating severity at the end of the 12-week treatment period, compared with a 31% reduction for patients receiving placebo. Likewise, approximately one-third of patients treated with linaclotide reported ≥ 50% reduction in abdominal bloating over the 12-week treatment period, compared with 18% of patients treated with placebo. A ≥ 30% reduction from baseline in abdominal bloating for at least 6 of the 12 treatment-period weeks (an endpoint analogous to the FDA-recommended abdominal pain endpoint for IBS-C)[22] was reported in more than 40% of patients treated with linaclotide, compared with 29% of patients treated with placebo. These results are particularly important, given the lack of available CIC treatments that improve abdominal bloating.

The etiology of abdominal bloating is not well understood; however, visceral hypersensitivity and alterations in intestinal gas production or transit both appear to be potential mechanisms.[23] It is possible that linaclotide improves abdominal bloating by accelerating colonic transit, thereby improving the evacuation of stool and gas, and by acting on colonic nociceptors to decrease afferent signaling via the active transport of cGMP across the basolateral surface of enterocytes.[15] Future studies will be required to elucidate the precise mechanisms by which linaclotide improves abdominal bloating. In particular, recent advancements in GI functional magnetic resonance imaging (fMRI) techniques may allow for further insight to the distribution of intestinal contents, including gas, and improvement in symptoms.[24] Future studies utilizing active comparators (e.g., laxatives) rather than placebo could also provide insight into the effect of linaclotide on abdominal bloating independent of its effect on constipation.

Abdominal distention (or girth) was not assessed in this trial due to the significant complexity and burden of accurately measuring abdominal girth. Although there are no data in CIC patients, IBS patients with subjective sensations of abdominal bloating without visible abdominal distention have lower rectal thresholds to balloon distention than IBS patients with both bloating and distention.[23] Patients with abdominal distention appear to have abnormalities in the abdominal accommodation reflex secondary to paradoxical relaxation of the anterior abdominal wall and a contraction of the diaphragm.[25] Further study is needed to determine whether linaclotide improves abdominal distention.

Recent research suggests that CIC and IBS-C are part of a disease spectrum, with significant abdominal symptoms present in patients with CIC.[26–28] Rome criteria, while useful as a diagnostic tool, may not effectively distinguish between these 2 conditions.[29] Patients enrolled in this trial met Rome II criteria for functional constipation (i.e., CIC) and had significant abdominal bloating; patients were excluded if they reported abdominal pain or abdominal discomfort related to a change in stool frequency or appearance (i.e., Rome criteria for IBS). Presumably, some patients may have characterized bloating as abdominal discomfort during the pretreatment period and were therefore excluded from trial participation. Eligibility criteria of future trials of CIC patients with abdominal bloating should clearly distinguish abdominal bloating from abdominal discomfort.

Earlier phase 3 CIC trials of linaclotide did not require patients to meet a minimum threshold of abdominal bloating and, in fact, patients had only mild to moderate abdominal bloating at baseline (average patient-assessed score of 2.7–2.8 on a 1- to 5-point ordinal scale where 1 = none, 2 = mild, 3 = moderate, 4 = severe, and 5 = very severe).[18] Despite the low baseline abdominal bloating severity, linaclotide significantly improved abdominal bloating over the 12-week treatment period (decreases in the two trials of 0.4 and 0.5 in patients treated with linaclotide 145 μg, and of 0.5 and 0.4 in patients treated with linaclotide 290 μg) compared to placebo (decreases of 0.2 in both trials) (*P* < 0.01).[18] The current trial aimed to provide further assessments of abdominal bloating and required patients to have moderate to severe abdominal bloating, defined as an average patient-assessed score of ≥ 5 on a 0- to 10-point NRS, at baseline. The NRS was adopted in the current trial to be consistent with the scale used to measure abdominal bloating in the IBS-C phase 3 linaclotide trials,[30–31] to reflect the FDA requirement for measurement of abdominal pain in IBS-C trials,[22] and to be more consistent with methods of measuring somatic and neuropathic pain.[32] A score of 5 on the 11-point NRS roughly corresponds to moderate bloating (i.e., a score of 3) on the 5-point scale used in the previous linaclotide CIC trials. In the current trial, the average baseline abdominal bloating score was 7.1, which falls roughly within the moderate to severe range on the previous 5-point scale. Significant improvements from baseline in patient ratings of abdominal bloating were seen over the 12-week treatment period in the linaclotide dose groups (decreases of 2.5 in both groups) compared to placebo (decrease of 1.6) (*P* < 0.001). The sum of the data from these three large, phase 3 trials supports the conclusion that linaclotide is a safe and efficacious agent for the treatment of CIC. This study, in particular, provides support that linaclotide effectively treats bowel symptoms and abdominal bloating in CIC patients with moderate to severe abdominal bloating.

The objective of this trial was to evaluate the efficacy and safety of linaclotide in patients with CIC and moderate-to-severe abdominal bloating. The study sponsors (Forest Laboratories—a subsidiary of Actavis Inc., and Ironwood Pharmaceuticals) selected the 9 of 12 week CSBM 3+1 responder over a bloating-specific primary endpoint as the former is the primary endpoint used in the two pivotal Phase 3 registration trials of linaclotide. Future studies designed and powered specifically to assess abdominal bloating and which follow patients for longer than 12 weeks may be warranted to determine the long-term efficacy and safety of linaclotide for the management of abdominal bloating in CIC patients.

With regard to AEs reported in the current trial, diarrhea occurred less frequently with the lower dose of linaclotide (i.e., 145 μg) compared with the higher dose (290 μg). At the 290 μg dose of linaclotide, diarrhea AE rates were similar in the current study and the two previous pivotal trials (17% in the current trial versus 14% in the pooled pivotal trials).[18] However, diarrhea was less commonly reported by those randomized to linaclotide 145 μg in the current trial (6%) compared to the initial pivotal trials (16%). In the current trial, the incidence of discontinuation from the study due to an AE of diarrhea was 1.3% and 5.0% in the 145 μg and 290 μg dose groups, respectively (and 0.6% in the placebo group); in the pivotal trials, there was less difference between linaclotide dose groups in the incidence of diarrhea AEs leading to study discontinuation (4.7% and 3.8% in the 145 μg and 290 μg dose groups, respectively; 0.5% in the placebo group). The reason for the differences in reported incidence of diarrhea AEs, both between linaclotide doses in the current trial and between the current trial and the previous pivotal trials, is unknown. It is important to note that SAEs were infrequent in the current trial and, as in the pivotal trials, diarrhea was not reported as an SAE.[18]

In conclusion, this trial has demonstrated that linaclotide, a GC-C agonist, has a consistent effect on bowel symptoms and significantly improves abdominal bloating in CIC patients with moderate to severe baseline abdominal bloating. This finding is important given the dearth of agents available to treat abdominal bloating in patients with CIC.

## Supporting Information

S1 CONSORT Checklist CONSORT Checklist. (DOCX)

S1 Table Other Secondary and Additional Efficacy Results During the 12-week Treatment Period (ITT Population). (DOCX)
