# Supplementary material for: Linaclotide in Chronic Idiopathic Constipation Patients with Moderate to Severe Abdominal Bloating: A Randomized, Controlled Trial
Source: PLoS One. 2015 Jul 29;10(7):e0134349. doi: 10.1371/journal.pone.0134349 (PMC4519259; doi:10.1371/journal.pone.0134349)
Supplement: S1 Table — (DOCX) [file pone.0134349.s002.docx]

**S1 Table. Other Secondary and Additional Efficacy Results During the 12-week Treatment Period (ITT Population)**

|  | **Placebo (N=171)** | **Linaclotide** | | | |
| --- | --- | --- | --- | --- | --- |
|  |  | **145 µg (N=153)** | ***P* value** | **290 µg (N=159)** | ***P* value** |
| **Abdominal Bloating** (11-point NRS) |  |  |  |  |  |
| Distribution of % change from baseline at week 12 ^a^ | N/A | N/A | 0.0054** | N/A | 0.0012** |
| % of patients with ≥ 1-point weekly mean decrease in bloating for ≥ 6/12 weeks ^b^ | 36.8 | 53.6 | 0.0023* | 50.9 | 0.0107* |
| Change from baseline in % of days with abdominal bloating < 3, mean ^c,d^ | 15.6 | 25.3 | 0.0092* | 25.6 | 0.0055* |
| **CSBMs** |  |  |  |  |  |
| Change from baseline in CSBMs/week at week 1, mean ^e^ | 0.6 | 2.0 | <0.0001** | 1.7 | 0.0004* |
| Change from baseline in CSBMs/week at week 4, mean ^e^ | 1.0 | 2.7 | <0.0001** | 2.6 | <0.0001* |
| Change from baseline in CSBMs/week at week 8, mean ^e^ | 1.2 | 2.7 | 0.0001** | 2.2 | 0.0099* |
| Change from baseline in CSBMs/week at week 12, mean ^e^ | 1.0 | 2.2 | 0.0002** | 2.4 | <0.0001* |
| **SBMs** |  |  |  |  |  |
| Change from baseline in SBMs/week at week 1, mean ^e^ | 1.9 | 4.2 | <0.0001** | 4.1 | <0.0001* |
| Change from baseline in SBMs/week at week 4, mean ^e^ | 1.5 | 4.1 | <0.0001** | 3.9 | <0.0001* |
| Change from baseline in SBMs/week at week 8, mean ^e^ | 1.6 | 3.6 | <0.0001** | 3.7 | <0.0001* |
| Change from baseline in SBMs/week at week 12, mean ^e^ | 1.4 | 3.1 | <0.0001** | 3.0 | <0.0001* |
| **Stool Consistency** (7-point BSFS) |  |  |  |  |  |
| Change from baseline at week 12, mean ^e^ | 0.8 | 2.1 | <0.0001** | 2.3 | <0.0001* |
| **Straining** (5-point ordinal scale) |  |  |  |  |  |
| Change from baseline at week 12, mean ^e^ | -0.9 | -1.6 | <0.0001** | -1.7 | <0.0001* |
| **Abdominal Discomfort** (11‑point NRS) |  |  |  |  |  |
| Mean abdominal discomfort score ^c^ | 5.0 | 4.1 |  | 4.3 |  |
| Change from baseline, mean ^c,e^ | -1.7 | -2.5 | 0.0002* | -2.4 | 0.0013* |
| % change from baseline, mean ^c,e^ | -26.2 | -38.1 | 0.0009* | -36.0 | 0.0057* |
| **Abdominal Pain** (11‑point NRS) |  |  |  |  |  |
| Mean abdominal pain score ^c^ | 4.0 | 3.3 |  | 3.3 |  |
| Change from baseline, mean ^c,e^ | -1.6 | -2.4 | 0.0008* | -2.3 | 0.0027* |
| % change from baseline, mean ^c,e^ | -33.1 | -39.6 | 0.2656* | -42.7 | 0.0976* |
| **Abdominal Fullness** (11‑point NRS) |  |  |  |  |  |
| Mean abdominal fullness score ^c^ | 5.6 | 4.7 |  | 4.8 |  |
| Change from baseline, mean ^c,e^ | -1.6 | -2.6 | <0.0001* | -2.5 | 0.0003* |
| **Abdominal Cramping** (11‑point NRS) |  |  |  |  |  |
| Mean abdominal cramping score ^c^ | 3.8 | 3.1 |  | 3.2 |  |
| Change from baseline, mean ^c,e^ | -1.5 | -2.2 | 0.0018* | -2.1 | 0.0035* |
| **Adequate Relief of CIC Symptoms** |  |  |  |  |  |
| % of patients reporting adequate relief for ≥ 9/12 weeks ^b^ | 16.4 | 33.3 | 0.0005* | 35.2 | <0.0001* |
| **Degree of Relief of Constipation Symptoms** (7-point balanced scale) |  |  |  |  |  |
| % of patients reporting degree of relief ≤ 3 for all weekly scores *or* ≤ 2 for ≥ 6/12 weeks ^b^ | 19.9 | 36.6 | 0.0008* | 37.1 | 0.0005* |

ANCOVA = analysis of covariance; BSFS = Bristol Stool Form Scale; CIC = chronic idiopathic constipation; CSBM = complete SBM; ITT = intent to treat; NRS = numerical rating scale; SBM = spontaneous bowel movement.

** Secondary endpoint (nominal *P* value); * Additional endpoint

^a^ *P* values were based on a comparison of linaclotide vs. placebo using a Kolmogorov-Smirnov test for equality of distribution.

^b^ *P* values were based on a comparison of linaclotide vs. placebo using a Cochran‑Mantel‑Haenszel test controlling for geographic region.

^c^ Means are over the 12-week treatment period.

^d^ Changes from baseline are the arithmetic means; *P* values were based on a comparison of linaclotide vs. placebo using rank-transformed normal scores in an ANCOVA model with treatment group and geographic region as factors and corresponding baseline value as a covariate.

^e^ Changes from baseline are the least-squares means from an ANCOVA model; *P* values were based on a comparison of linaclotide vs. placebo using an ANCOVA model with treatment group and geographic region as factors and corresponding baseline value as a covariate.
